# Supplementary material for: Prospective validation of smartphone-based heart rate and respiratory rate measurement algorithms
Source: Commun Med (Lond). 2022 Apr 12;2:40. doi: 10.1038/s43856-022-00102-x (PMC9053269; doi:10.1038/s43856-022-00102-x)
Supplement: Supplementary file 1 — Supplementary Information [file 43856_2022_102_MOESM1_ESM.pdf]

Supplementary Information

Supplementary Methods

Additional heart rate algorithm details

A temporal ensemble model was used to improve the robustness of the heart rate algorithm. Each new RGB input frame results in computing a temporally localized heart rate estimate and the corresponding SNR value using the Fast Fourier Transform (FFT). The heart rate values estimated throughout a measurement session are averaged using the corresponding SNR values as weights. This approach enabled our algorithm to make accurate estimates even when high SNR measurements are made only in some parts of the session.

In a naive implementation of a spatial ensemble model, the computational cost grows linearly with the number of weak predictors within the ensemble and with the computational cost to process each weak predictor (proportional to the size of the ROI). This presents a challenge for real-time applications on a mobile processor since the time budget to process each input frame is limited to tens of milliseconds (e.g. 66.7ms at 15 Hz sampling rate). We reduced the marginal cost of adding another weak predictor to a sub-millisecond range (independent of the size of ROIs) by using the integral image representation, similar to the Viola-Jones object detection model. The speedup comes from reducing the computational time complexity of computing the RGB mean of an additional arbitrary rectangular ROI from linear to constant. A finger presence classifier based on RGB color statistics was developed to reject non-relevant input frames. The mean and variance threshold values for the classifier were empirically determined.

Supplementary Figures

Supplementary Figure 1. Detailed user interface for the algorithms.

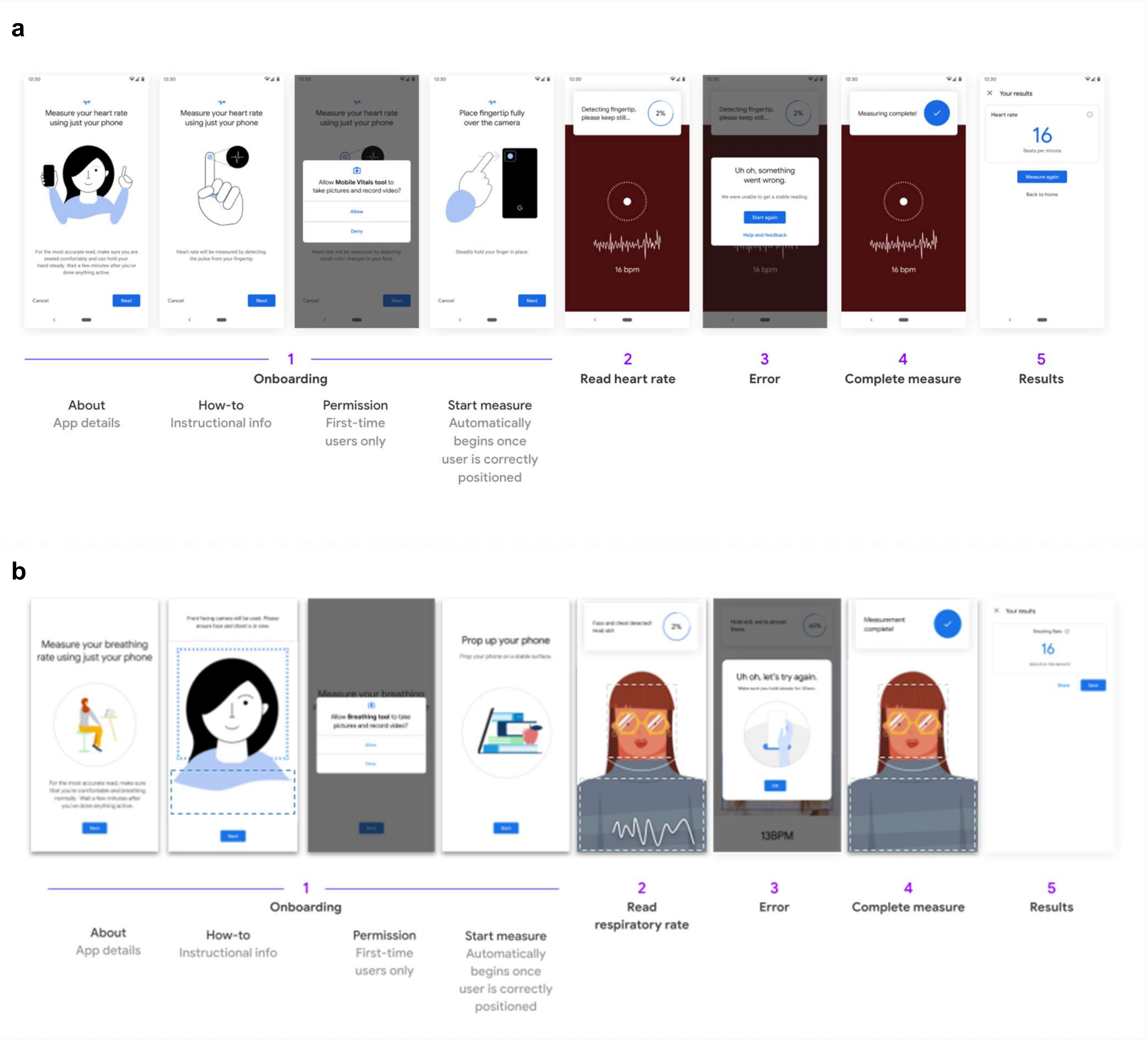

a, interface for the heart rate (HR) algorithm and **b**, and interface for the respiratory rate (RR) algorithm.

Supplementary Figure 2. Number of participants enrolled and data analyzed.

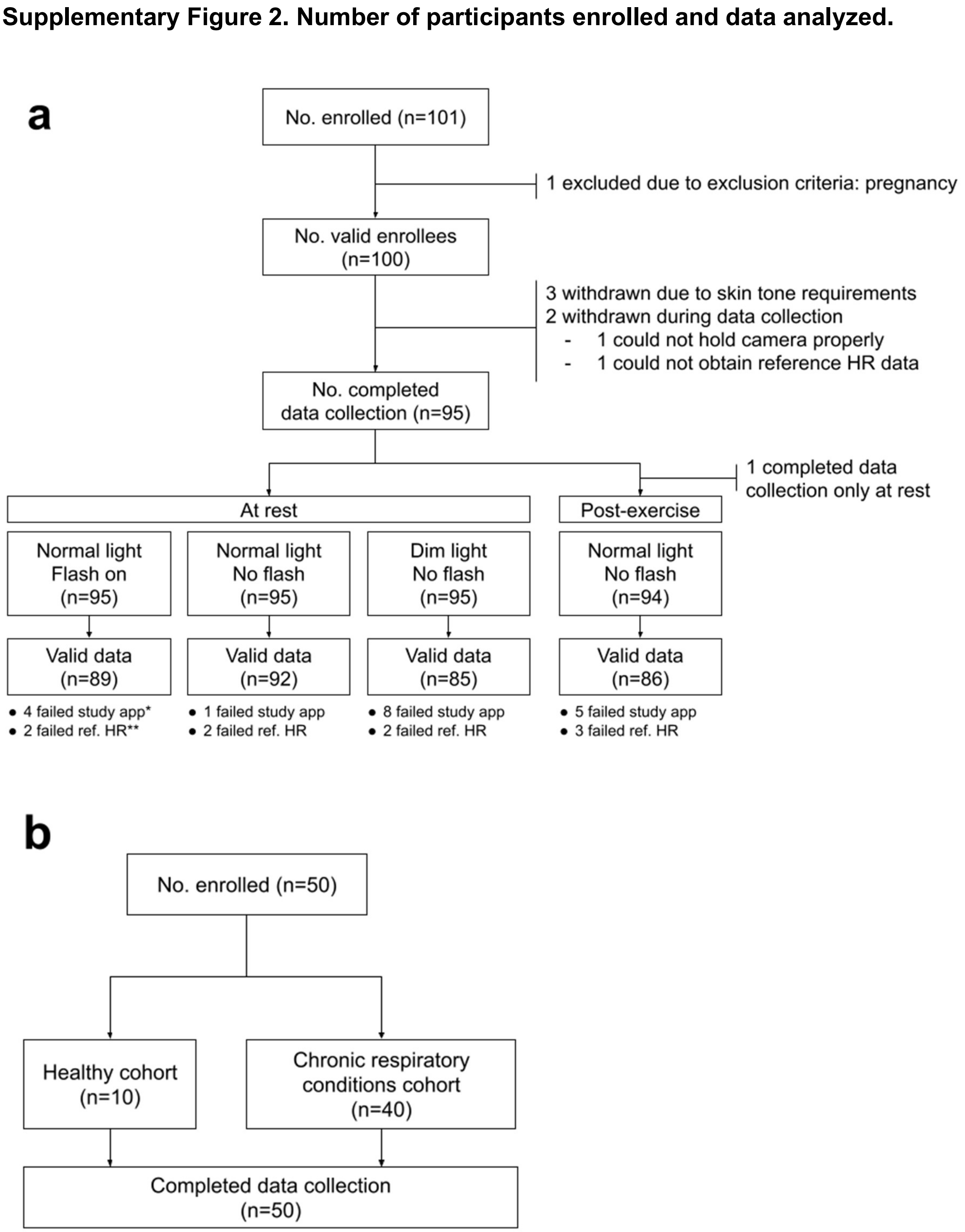

Flowcharts are show for **a**, the heart rate (HR) study and **b**, the respiratory rate (RR) study.

*Failed study application data collection due to signal-to-noise ratio <0; **Failed reference HR collection.

Supplementary Figure 3. Additional Bland-Altman plots for the heart rate (HR) study.

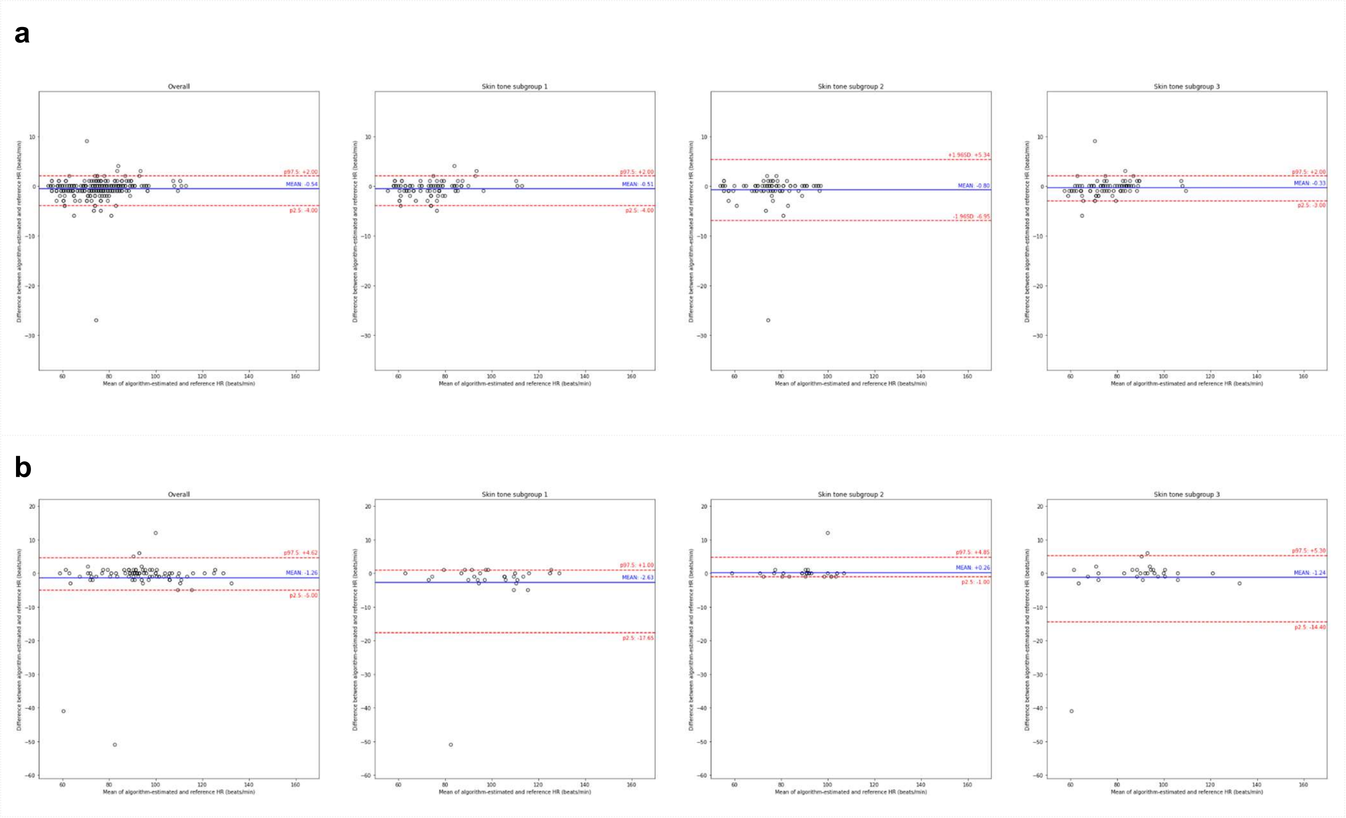

Results are presented for participants (**a**) at rest and (**b**) post-exercise. Left to right: plots from the full study followed by subgroups based on skin type (see Figure 1). The reference HR was obtained from a pulse oximeter (see Methods). Dots represent individual participants; blue lines indicate the mean difference; red lines indicate the limits of agreement (based on percentiles or mean and standard deviation; see labels in the plot).

Supplementary Figure 4. Participants' survey results after the respiratory rate (RR) study.

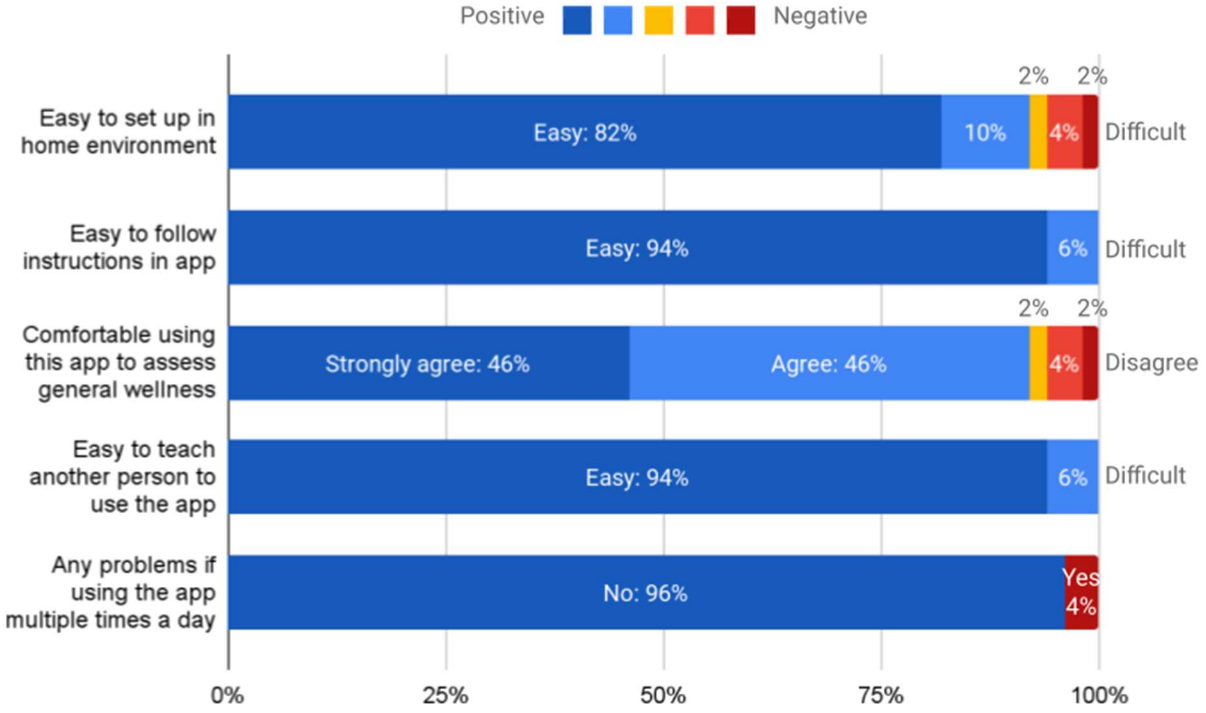

The exact survey questions are provided in Supplementary Table 7.

Supplementary Tables

Supplementary Table 1a. Eligibility criteria of the heart rate (HR) study.

**Inclusion criteria**	● Age > 18 years<br>● Participant is able and willing to provide written informed consent (Attached document: GH-VV-002 [ICF Heart Rate Study])<br>● Reads and speaks English. (Spanish speaking participants may be enrolled based on IRB requirements and/or availability of bilingual clinical study staff)
**Exclusion criteria**	● Unwilling or unable to remove makeup or face covering for study participation<br>● Pregnancy - Women who are, or believe they might be, pregnant<br>● Inability to understand study procedures and/or the informed consent process<br>● Other medical complications that preclude completion of the study as determined by the principal investigator at each study site<br>● Significant tremor that is present while seated at time of study as determined by the study coordinator (upper chest or face)<br>● Ineligibility for physical activity based on interpretation of PAR-Q by the site's principal investigator. Study participants who answer yes to one or more of the PAR-Q questions should be further assessed for eligibility by the site's principal investigator.
**Enrollment requirements**	**Age**<br>● ≥25% between 20–39 years old<br>● ≥25% between 40–59 years old<br>● ≥25% greater than 60 years old<br><br>**Skin Tones**<br>Measured by Pantone Device; see Supplementary Table 2 for details<br>● ≥30% Very light, light, intermediate (minimum 10 participants from each group, maximum 40 participants in total)<br>● ≥20% Tan, brown (minimum 10 participants from each group, maximum 30 participants in total)<br>● ≥30% Dark (minimum 30 participants)

Supplementary Table 1b. Eligibility criteria of the respiratory rate (RR) study.

**Inclusion criteria**	● Age > 18 years<br>● Participant is able and willing to provide written informed consent<br>● Reads and speaks English. (Spanish speaking participants may be enrolled based on IRB requirements and/or availability of bilingual clinical study staff)	
	Healthy cohort (n=10)	● No respiratory symptoms at time of study and no history of chronic respiratory conditions.
	Cohort of chronic respiratory conditions (n=40)	● COPD of moderate severity as defined by at least one of the following:<br>○ hospitalized for exacerbation within 1 year<br>○ received oral steroids for COPD within 3 months<br>○ Current use of supplemental oxygen at home.<br>AND<br>○ A Modified Medical Research Council (mMRC) breathlessness scale score of > 26<br>● Asthma that is not currently well-controlled, as determined by by one or more of the following;<br>○ Emergency department visit and/or hospital admission for asthma within the preceding 3 months<br>○ Use of oral corticosteroids for > 3 days for treatment of asthma within the preceding 3 months<br>○ Use of a short acting beta agonist (SABA) for > 2 days during the preceding 7 days
**Exclusion criteria**	● Pregnancy - Women who are, or believe they might be, pregnant<br>● Inability to understand study procedures and/or the informed consent process<br>● Medical or other complications that preclude completion of the study as determined by the principal investigator at each study site<br>● Significant tremor that is present while seated at time of study as determined by the study coordinator (upper chest or face)	
**Enrollment requirements**	Age<br>● > 20% between 20–39 years old<br>● > 20% between 40–59 years old<br>● > 40% greater than 60 years old<br>Race/Ethnicity<br>● > 20% self-identifying as of Hispanic/Latino ethnicity<br>● > 20% self-identifying as White<br>● > 20% self-identifying as African American/Black	

Supplementary Table 2. Objective skin tone information was objectively measured by applying a Pantone RM200QC Spectro to each participant's cheek and mapping the parameters to a Fitzpatrick skin type.

°ITA	Skin Tone Classification	Mapped to Fitzpatrick Skin Type
°ITA > 55°	Very Light	1
41° < °ITA ≤ 55°	Light	2
28° < °ITA ≤ 41°	Intermediate	3
10° < °ITA ≤ 28°	Tan	4
-30° < °ITA ≤ 10°	Brown	5
°ITA ≤ -30°	Dark	6

The numerical parameters were in the CIELAB color space, and the L* and b* parameters were converted to an individual typology angle (°ITA) as defined by the function [arctan(L* - 50)/b*] x 180/3.14159.

Supplementary Table 3. Participant race and skin tone group stratified by signal-to-noise ratio (SNR) readings < 0 for the heart rate (HR) study.

	No. of participants with SNR<0	No. of participants with SNR≥0	P value*
Age	45.5 ± 16.4	41.2 ± 14.7	0.372
Sex*			
Female	11 (15.4%)	60 (84.5%)	>0.99
Male	3 (12.5%)	21 (87.5%)	
Race/ethnicity*			
White, non-Hispanic	5 (20.0%)	20 (80.0%)	0.798
Black, non-Hispanic	8 (13.1%)	53 (86.9%)	
Asian / pacific islander	1 (14.3%)	6 (85.7%)	
Multiple races, non-Hispanic	0 (0.0%)	1 (100%)	
Multiple races, Hispanic	0 (0.0%)	1 (100%)	
Measured skin tone**			
1 (Fitzpatrick types 1-3)	5 (16.1%)	26 (83.9%)	0.938
2 (Fitzpatrick types 4-5)	4 (12.5%)	28 (87.5%)	
3 (Fitzpatrick type 6)	5 (15.6%)	27 (84.4%)	
Fitzpatrick type			
1 (Very light)	0 (0.0%)	1 (100%)	0.194
2 (Light)	1 (6.3%)	15 (93.8%)	
3 (Intermediate)	4 (28.6%)	10 (71.4%)	
4 (Tan)	2 (40.0%)	3 (60.0%)	
5 (Brown)	2 (7.4%)	25 (92.6%)	
6 (Dark)	5 (15.6%)	27 (84.4%)	

* P values were calculated by Fisher's exact test comparing participants with SNR<0 and those with SNR≥0

** Fitzpatrick determination based on measurement with Pantone device and conversion to Fitzpatrick scale

Supplementary Table 4. Detailed results of the heart rate (HR) study.

Session	Number of participants	Number of data points	Reference HR (beats/min)		MAE (beats/min)	MAPE		
			Mean ± SD	Range	Mean ± SD	Mean ± SD	Median (IQR)	95th percentile
Total population	95	352	79.8 ± 14.6	54–134	1.32 ± 3.92	1.63 ± 4.27	1.14 (0.0–1.64)	4.84
At rest, flash on, regular light	95	89	75.6 ± 11.1	55–113	0.87 ± 1.06	1.16 ± 1.38	1.22 (0.0–1.47)	3.97
At rest, flash off, regular light	95	92	74.8 ± 11.0	54–111	1.23 ± 3.01	1.64 ± 3.60	1.20 (0.0–1.64)	4.77
At rest, flash off, dim light	95	85	75.6 ± 11.2	55–110	0.96 ± 1.26	1.33 ± 1.77	1.18 (0.0–1.67)	5.19
Post-exercise	94	86	93.9 ± 15.4	59–134	2.23 ± 7.05	2.39 ± 7.45	1.02 (0.0–1.75)	5.42
Subgroup 1: very light, light, and intermediate skin tone	31	119	81.0 ± 17.4	56–129	1.53 ± 4.73	1.77 ± 4.46	1.27 (0.0–1.83)	4.84
At rest, flash on, regular light	31	30	74.5 ± 11.8	58–113	1.03 ± 1.16	1.40 ± 1.56	1.28 (0.0–1.69)	4.49
At rest, flash off, regular light	31	31	74.0 ± 11.6	58–111	1.00 ± 1.15	1.36 ± 1.57	1.27 (0.0–1.64)	4.59
At rest, flash off, dim light	31	28	74.2 ± 12.3	56–110	1.04 ± 1.23	1.43 ± 1.72	1.23 (0.0–1.74)	5.13
Post-exercise	31	30	101.0 ± 15.9	63–129	3.03 ± 9.15	2.90 ± 8.46	1.09 (0.82–2.09)	4.36
Subgroup 2: tan and brown skin tone	32	113	78.8 ± 12.7	54–107	1.04 ± 2.87	1.32 ± 3.30	0.98 (0.0–1.37)	4.86
At rest, flash on, regular light	32	28	76 .0± 11.3	55–97	0.86 ± 1.15	1.14 ± 1.40	1.23 (0–1.43)	2.32
At rest, flash off, regular light	32	29	75.2 ± 11.4	54–96	1.59 ± 4.98	1.97 ± 5.68	0.0 (0.0–1.35)	4.93
At rest, flash off, dim light	32	29	75 .0± 11.3	55–96	0.86 ± 1.27	1.20 ± 1.78	0.0 (0.0–1.72)	5.35
Post-exercise	32	27	89.5 ± 11.3	59–107	0.85 ± 2.28	0.93 ± 2.43	0.0 (0.0–1.10)	1.35
Subgroup 3: dark skin tone	32	120	79.7 ± 13.3	58–134	1.37 ± 3.89	1.77 ± 4.87	1.15 (0.0–1.64)	4.48
At rest, flash on, regular light	32	31	76.2 ± 10.6	61–110	0.71 ± 0.86	0.95 ± 1.17	0.91 (0–1.49)	3.24
At rest, flash off, regular light	32	32	75.3 ± 10.4	58–108	1.12 ± 1.66	1.61 ± 2.50	1.22 (0–1.93)	3.71
At rest, flash off, dim light	32	28	77.5 ± 10.0	60–107	1.00 ± 1.31	1.35 ± 1.87	1.19 (0–1.41)	3.99
Post-exercise	31	29	90.6 ± 15.9	61–134	2.69 ± 7.51	3.22 ± 9.27	1.12 (0.0–2.17)	6.27

Abbreviations: HR, heart rate; MAE, mean absolute error; MAPE, mean absolute percentage error; SD, standard deviation; IQR, interquartile ranges

Supplementary Table 5. Detailed results of the respiratory rate (RR) study.

Subgroups	N	Reference RR (breaths/min)		MAE (breaths/min)		
		Mean ± SD	Range	Mean ± SD	Range	P value
Algorithm version A	50	15.5 ± 3.6	8–22	0.84 ± 0.97	0–6	<0.001
Healthy	10	16.0 ± 3.7	10–20	0.60 ± 0.52	0–1	0.001
Chronic respiratory conditions	40	15.4 ± 3.6	8–22	0.90 ± 1.05	0–6	<0.001
Algorithm version B	50	15.3 ± 3.7	8–26	0.78 ± 0.61	0–2	<0.001
Healthy	10	16.4 ± 3.7	12–22	0.70 ± 0.67	0–2	<0.001
Chronic respiratory conditions	40	15.1 ± 3.7	8–26	0.80 ± 0.60	0–2	0.001

Abbreviations: RR, respiratory rate; MAE, mean absolute error; SD, standard deviation

Supplementary Table 6. Subgroup analysis for algorithm version B of the respiratory rate (RR) study.

Subgroups	N	Reference RR (breaths/min)		MAE (breaths/min)		
		Mean ± SD	Range	Mean ± SD	Range	P value
Age subgroups						
<40 years old	17	15.8 ± 4.4	10–26	0.79 ± 0.64	0–2	<0.001
40–59 years old	21	15.3 ± 3.1	10–22	0.79 ± 0.60	0–2	<0.001
≥60 years old	12	14.8 ± 3.8	8–22	0.75 ± 0.62	0–2	<0.001
Race/ethnicity						
White, non-Hispanic	18	15.4 ± 4.1	8–26	0.72 ± 0.67	0–2	<0.001
Black, non-Hispanic	6	18.0 ± 4.0	12–22	0.83 ± 0.41	0–1	<0.001
Hispanic/Latino ethnicity	23	14.5 ± 2.6	10–19	0.83 ± 0.63	0–2	<0.001
Other	3	16.3 ± 6.8	12–24	0.67± 0.58	0–1	0.010

Supplementary Table 7. Participant experience survey on the respiratory rate (RR) measurement algorithm.

How easy do you believe it would be to find a place to prop the phone up at home?	☐ Easy*	☐ Somewhat Easy*	☐ Neither easy nor difficult	☐ Somewhat difficult	☐ Difficult
How would you rate the experience of following instructions in this application?	☐ Easy*	☐ Somewhat Easy*	☐ Neither easy nor difficult	☐ Somewhat difficult	☐ Difficult
How much do you agree with the following statement, I would feel comfortable using this application to assess my general wellness?	☐ Strongly agree*	☐ Agree*	☐ Neither agree nor disagree	☐ Disagree	☐ Strongly disagree
How well do you feel you could teach another person to use this application?	☐ Easy*	☐ Somewhat Easy*	☐ Neither easy nor difficult	☐ Somewhat difficult	☐ Difficult
If you needed to use this app several times per day do you think there would be any problems?	☐ Yes	☐ No*			
(Optional) Do you have any other feedback you'd like to give on this application?	(Free description)				

* These responses were considered a positive response, and excludes neutral responses: “neither easy nor difficult” and “neither agree nor disagree”.
