# Supplementary material for: Prospective validation of smartphone-based heart rate and respiratory rate measurement algorithms
Source: Commun Med (Lond). 2022 Apr 12;2:40. doi: 10.1038/s43856-022-00102-x (PMC9053269; doi:10.1038/s43856-022-00102-x)
Supplement: Supplementary file 3 — Reporting Summary [file 43856_2022_102_MOESM3_ESM.pdf]

nature research

Corresponding author(s): Si-Hyuck Kang

Last updated by author(s): Jan 30, 2022

Reporting Summary

Nature Research wishes to improve the reproducibility of the work that we publish. This form provides structure for consistency and transparency in reporting. For further information on Nature Research policies, see our Editorial Policies and the Editorial Policy Checklist.

Statistics

For all statistical analyses, confirm that the following items are present in the figure legend, table legend, main text, or Methods section.

n/a	Confirmed	
☐	☒	The exact sample size (n) for each experimental group/condition, given as a discrete number and unit of measurement
☐	☒	A statement on whether measurements were taken from distinct samples or whether the same sample was measured repeatedly
☐	☒	The statistical test(s) used AND whether they are one- or two-sided<br>*Only common tests should be described solely by name; describe more complex techniques in the Methods section.*
☐	☒	A description of all covariates tested
☐	☒	A description of any assumptions or corrections, such as tests of normality and adjustment for multiple comparisons
☐	☒	A full description of the statistical parameters including central tendency (e.g. means) or other basic estimates (e.g. regression coefficient) AND variation (e.g. standard deviation) or associated estimates of uncertainty (e.g. confidence intervals)
☐	☒	For null hypothesis testing, the test statistic (e.g. F, t, r) with confidence intervals, effect sizes, degrees of freedom and P value noted<br>*Give P values as exact values whenever suitable.*
☒	☐	For Bayesian analysis, information on the choice of priors and Markov chain Monte Carlo settings
☒	☐	For hierarchical and complex designs, identification of the appropriate level for tests and full reporting of outcomes
☒	☐	Estimates of effect sizes (e.g. Cohen's d, Pearson's r), indicating how they were calculated

Our web collection on statistics for biologists contains articles on many of the points above.

Software and code

Policy information about availability of computer code

Data collection	Google Drive was used for data collection.
Data analysis	Python 3.8 and R programming were used for data analysis.

For manuscripts utilizing custom algorithms or software that are central to the research but not yet described in published literature, software must be made available to editors and reviewers. We strongly encourage code deposition in a community repository (e.g. GitHub). See the Nature Research guidelines for submitting code & software for further information.

Data

Policy information about availability of data

All manuscripts must include a data availability statement. This statement should provide the following information, where applicable:

- Accession codes, unique identifiers, or web links for publicly available datasets
- A list of figures that have associated raw data
- A description of any restrictions on data availability

The data are not publicly available because they contain videos of the participants and public release was not part of informed consent.

Field-specific reporting

Please select the one below that is the best fit for your research. If you are not sure, read the appropriate sections before making your selection.

☒ Life sciences ☐ Behavioural & social sciences ☐ Ecological, evolutionary & environmental sciences

For a reference copy of the document with all sections, see nature.com/documents/nr-reporting-summary-flat.pdf

Life sciences study design

All studies must disclose on these points even when the disclosure is negative.

Sample size	95 participants in the HR study<br>50 participants in the RR study
Data exclusions	HR study: A total of 101 participants were enrolled. Study eligibility criteria are described in Methods. After excluding one participant who was found to have an exclusion criterion (pregnancy), there were 100 valid enrollees. Among these, 3 were withdrawn due to skin tone distribution requirements, and 2 were withdrawn during data collection due to difficulty in data collection (failure to follow the instruction on holding the phone and a health condition that prevents us from collecting reference HR). Thus, 95 participants completed data collection.<br>RR study: no data were excluded.
Replication	This was a human study, which cannot be replicated.
Randomization	Not relevant because it was a prospective observational study
Blinding	Not relevant because participants were not allocated to different study groups

Reporting for specific materials, systems and methods

We require information from authors about some types of materials, experimental systems and methods used in many studies. Here, indicate whether each material, system or method listed is relevant to your study. If you are not sure if a list item applies to your research, read the appropriate section before selecting a response.

Materials & experimental systems

n/a	Involved in the study	
☒	☐	Antibodies
☒	☐	Eukaryotic cell lines
☒	☐	Palaeontology and archaeology
☒	☐	Animals and other organisms
☐	☒	Human research participants
☒	☐	Clinical data
☒	☐	Dual use research of concern

Methods

n/a	Involved in the study	
☒	☐	ChIP-seq
☒	☐	Flow cytometry
☒	☐	MRI-based neuroimaging

Human research participants

Policy information about studies involving human research participants

Population characteristics	HR study: participants from diverse populations in terms of objectively measured skin tones, ranging from very light to dark skin<br>RR study: participants with and without chronic respiratory conditions
Recruitment	Participants were enrolled at a clinical research site (Meridian, Savannah, GA).
Ethics oversight	The HR study protocol was approved by Advarra IRB (Columbia, MD; protocol no. Pro00046845).<br>The RR study protocol was approved by Aspire IRB (now WCG IRB, Puyallup, WA; protocol no. 20201594).

Note that full information on the approval of the study protocol must also be provided in the manuscript.
